# Association between Sleep Duration and 24-Hour Urine Free Cortisol in the MrOS Sleep Study

**DOI:** 10.1371/journal.pone.0075205

**Published:** 2013-09-27

**Authors:** Madhu N. Rao, Terri Blackwell, Susan Redline, Naresh M. Punjabi, Elizabeth Barrett-Connor, Thomas C. Neylan, Katie L. Stone, Osteoporotic Fractures in Men (MrOS) Study Group

**Affiliations:** 1 Division of Endocrinology, University of California San Francisco, San Francisco, California, United States of America; 2 Research Institute, California Pacific Medical Center, San Francisco, California, United States of America; 3 Department of Medicine, Brigham and Women’s Hospital and Beth Israel Deaconess Medical Center, Harvard Medical School, Boston, Massachusetts, Unites States of America; 4 Division of Pulmonary and Critical Care Medicine, Johns Hopkins University, Baltimore, Maryland, United States of America; 5 Department of Epidemiology, University of California San Diego, La Jolla, California, United States of America; 6 San Francisco VA Medical Center and Department of Psychiatry, University of California San Francisco, San Francisco, California, United States of America; University of Rochester, United States of America

## Abstract

**Context:**

Short sleep duration is associated with adverse health outcomes, but the mechanisms involved are unknown. It has been postulated that short sleep duration may elevate cortisol levels, but studies have had conflicting results. It is unclear whether these differing findings may be due to methodological issues, such as assessment of sleep duration. Specifically, objective versus subjective methods of measuring habitual sleep duration may account for the conflicting results found in epidemiological studies.

**Objective:**

Our goal was to determine whether habitual sleep duration, measured objectively (by actigraphy) and subjectively (by self-report), was associated with 24-hour urine free cortisol (UFC), a measure of integrated cortisol secretion. Our secondary goal was to determine whether slow wave sleep (SWS, determined by polysomnography) was associated with 24-hour UFC.

**Design/Setting:**

Cross sectional study of community dwelling older men.

**Patients/Participants:**

325 men (mean age = 76.6 years, SD = 5.5) from the Portland site of the MrOS Sleep Study, who underwent 24-hour urine collection, polysomnography, actigraphy and sleep questionnaire.

**Primary Outcome:**

24-hour UFC.

**Results:**

In this study of community dwelling older men, self-reported sleep duration was inversely related to 24-hour UFC levels. Participants reporting <5 hours of habitual sleep had an adjusted mean 24-hour UFC of 29.8 ug, compared to 28.0 ug in participants reporting >5 to <8 hours of sleep 25.5 ug in those reporting >8 hours of habitual sleep. However, sleep duration determined by actigraphy was not associated with 24-hour UFC in either univariable or multivariable regression models. SWS was not associated with 24-hour UFC.

**Conclusion:**

Objectively measured (i.e., actigraphic) sleep duration is not associated with 24-hour UFC in these community dwelling older men. This finding, together with prior studies, suggests that elevated levels of integrated cortisol secretion is not the mechanisms by which short sleep duration leads to adverse health outcomes.

## Introduction

Habitually short sleep duration is endemic in society today [1]. Over the past 50 years, sleep duration in the US has decreased by 1–2 hours per night and more than 50% of adults sleep less than 6 hours per night [1], [2]. Recent research has shown that sleep duration of less than 6 hours, on average, is associated with adverse health consequences, including impaired glucose tolerance, decreased immune function and increased risk of obesity [3]–[6]. However, the mechanism by which short sleep duration affects physiological function is still unclear. One possibility is that short sleep duration is a form of chronic stress. Consequently, the primary neuroendocrine mediator of stress, the HPA axis, may be activated, leading to elevated cortisol levels. Indeed, some studies of sleep restriction in a laboratory setting have found that sleep deprivation results in increased cortisol levels [7], [8]. However, other laboratory-based studies have reported that cortisol is unchanged or decreased [9]–[11]. One large cross-sectional study has shown that self-reported sleep duration is inversely associated with salivary cortisol levels [12]. Only one small study (of less than 100 subjects) has tried to answer the question of whether objectively measured sleep duration is associated with cortisol levels in a community based population [13]; the authors found no relation between actigraphic sleep time and morning cortisol levels. However, this study assessed cortisol level at a single time point (i.e., upon awakening), which is highly variable. It is unknown whether integrated cortisol levels (over 24 hours) are associated with objectively measured sleep duration.

Cortisol can be measured in several ways, and the ideal method is still unclear [14]. Because cortisol secretion varies widely throughout the day, single measurements are less informative [14]–[16]. Furthermore, because free cortisol (rather than bound) is the active component, any measurement method should quantify or estimate this fraction [17]. Lastly, the ideal cortisol measure in observational studies may be based in part on the research question, the outcomes being examined and the population under study [14].

The MrOS Sleep study included over 3000 community dwelling older men who underwent objective measurements of sleep at home (actigraphy and polysomnography) and answered a sleep questionnaire. A subset of the cohort had a 24-hour urine collection that was assayed for free cortisol. We chose 24-hour urine free cortisol (UFC) because it is an integrated measure of cortisol secretion over time, and because it is a measure of free cortisol levels. Because the MrOS Sleep study is a community based sample unselected for sleep history, area-under-curve measures of cortisol excretion may be most accurate. The goal of the current study was to assess the association between habitual sleep duration (measured by actigraphy and self-report) and an integrated measure of cortisol secretion (i.e., 24-hour UFC). Our secondary goal was to determine the association between pre-specified sleep architecture (specifically, SWS) and 24-hour UFC.

In this study, we had a unique opportunity to examine both actigraphic and self-reported sleep data *within the same population*, whereas prior studies had only one method for determining sleep duration (12,13). Other strengths of this study include the measurement of integrated cortisol secretion (rather than a single time point) and the use of a community dwelling population of subjects, which allows for a more realistic assessment of sleep and cortisol secretion.

## Methods

### Participants and Study Design

The Osteoporotic Fractures in Men Study (MrOS) is a cohort of 5994 community dwelling men, age 65 and older, who were enrolled between 2000–2002 at six clinical centers in the United States [18], [19]. The Outcomes of Sleep Disorders in Older Men Study (MrOS Sleep Study), was an ancillary study that enrolled 3,135 participants from 2003–2005 for a comprehensive sleep assessment. Participants at one site (Portland, Oregon) were asked to provide a single 24-hour urine collection; men with incontinence (loss of more than 1 cup a day of urine) were excluded. Participation was voluntary, with collection on 357 of the 513 participants (70%). 24-hour UFC could be determined for 342 men. Cortisol levels were missing for 15 participants due to: 7 had missing samples; 4 participants had a UFC values that could not be measured due to an interfering analyte; 4 participants had missing information on the total volume of urine collected. Of the 342 with 24-hour UFC data, all had information on self-reported sleep duration, 320 had actigraphic sleep duration and 334 had SWS data as measured by polysomnography (PSG). Among these 342 participants, 17 were excluded from the analysis; 2 were taking prednisone, which affects the measurement of UFC, and 15 had 24-hour UFC data that was pathologically elevated or low [20]–[22]. Due to concern about Cushing’s syndrome (in which cortisol levels are elevated) or adrenal insufficiency (in which cortisol levels are decreased) confounding the relationship between sleep and UFC, values that were consistent with these disorders were not included in these analyses. Thus, the final study sample consisted of 325 men in the UFC analysis. Compared to the other 2779 men in the MrOS Sleep Study with data for actigraphy or PSG, this subset of 325 men had similar values for self-reported and actigraphic sleep duration. The MrOS study protocol and ancillary studies were approved by the Institutional Review Boards at each of the participating field sites (University of Alabama at Birmingham; University of Minnesota; Stanford University; University of California, San Diego; Oregon Health and Science University; University of Pittsburgh) and written informed consent was obtained from each participant prior to enrollment.

### Data Collection

#### 24-hour urine collection

For the 24-hour urine collection, thymol crystal preservative was placed in a 4-liter container, followed by 70 mL of heavy mineral oil; final weight of the urine sample was measured. Participants were provided standardized instructions on performing 24-hour urine collections at home and a cooler with refrigerant gel packs for cold storage of the urine. Participants were asked to empty their bladder prior to the start of the 24-hour urine collection. For the next 24 hours all urine was collected, including the first morning void, in the urine collection container. Any urine sample that was lost was recorded. Samples were returned to the clinic within 48 hours of collection completion, where the 24-hour urine volume was measured and samples were stored at −20°C. An effort was made to have the 24-hour urine collection coincide with the actigraph recording. Urine collection and actigraphy coincided in most men (n = 280, 87%). Of those who did not coincide, 24-hour urine collection for 8 men was within 1 week of the actigraphy recording and was >2 weeks from the actigraphy recording for 16 men. Twenty-three men with 24-hour urine collection did not have usable actigraph data. In addition, 94% of men (n = 308) collected their 24-hour urine within 1 week of the in-home PSG recording.

#### 24-hour Urine Free Cortisol (UFC) assay

UFC was measured at Esoterix Laboratory (Calabassas Hills, CA) using high performance liquid chromatography tandem mass spectrometry. Urine creatinine was measured (using a Jaffe Reaction) to verify the accuracy of the24 hour urine collection. Each sample was measured once for UFC and creatinine. Seventeen samples were randomly chosen to be run in duplicate. The intra-assay coefficient of variation for UFC was 5.7%.

#### Assessment of habitual sleep duration

Participants were asked about habitual sleep duration with the question “On most nights, how many hours do you sleep each night?”, with data collected rounded up to the hour. Actigraphic sleep duration was measured using the SleepWatch-O (Ambulatory Monitoring, Inc., Ardsley, NY) worn continuously for a minimum of 5 nights (mean ± SD, 5.2±0.9 nights). Participants were instructed to wear the actigraph on their non-dominant wrist, to be removed only when bathing or during water sports. The actigraph detects movement via a piezoelectric beam. Actigraphy has been shown to provide a reliable estimate of sleep-wake patterns [23]. Data were collected in 3 modes but are reported here based on digital integration mode (i.e., proportional integration mode) [24]. ActionW-2 software (Ambulatory Monitoring, Inc., Ardsley, NY) was used to analyze the actigraphy data. Details of the actigraphy scoring algorithms used have been published elsewhere [25], [26]. Participants completed sleep diaries for the time period they wore the actigraph. The diaries included time into and time out of bed and times the actigraph was removed. This information was used in editing the actigraphy data files. Inter-scorer reliability for editing the actigraphy data files has been excellent in our group (intra-class coefficient = 0.95) and actigraphic sleep duration has been shown to have good concordance with sleep duration from PSG [27].

Variables estimated from actigraphy used in this analysis included: 1) sleep duration: the hours per night spent sleeping while in bed after lights off; 2) sleep efficiency (SE), a measure of sleep fragmentation: the percentage of time in bed after lights off spent sleeping; 3) wake after sleep onset (WASO), a measure of sleep fragmentation: minutes of wake after sleep onset during the in bed interval, with sleep onset defined as the point when the participant achieved a 20-minute continuous block of sleep after lights off. All variables from actigraphy reflect data averaged over all nights they wore the device.

#### PSG

In-home sleep studies were completed using unattended, portable PSG (Safiro, Compumedics, Inc.®). The recording montage was as follows: C_3_/A_2_ and C_4_/A_1_ electroencephalograms (EEG), bilateral electro-oculograms and a bipolar submental electromyogram to determine sleep status; thoracic and abdominal respiratory inductance plethysmography to determine respiratory effort; airflow (by nasal-oral thermocouple and nasal pressure cannula); finger pulse oximetry; electrocardiogram; body position (mercury switch sensor); and bilateral tibialis leg movements (piezoelectric sensors). Trained, certified staff members performed home visits to set-up the units, similar to the protocol used in the Sleep Heart Health Study [28]. After data were downloaded, the data were transferred to the Central Sleep Reading Center (Cleveland, OH) to be scored by certified research polysomnologists. The overall failure rate for the home sleep studies was less than 4% and more than 70% of the studies were graded as being of excellent or outstanding quality [28]. Sleep stages were scored using standard criteria [29]. Data from EEG leads C_3_ and C_4_ were used to score sleep stages 3 and 4, slow wave sleep, presented as the percent of total sleep time spent in SWS. The inter-scorer reliability of SWS was 0.96, with even higher levels of intra-scorer reliability [30].

#### Other covariates

All participants completed questionnaires at the time of the clinic visit, which included items about demographics, medical history, physical activity, smoking and alcohol use. Physical activity was assessed using the Physical Activity Scale for the Elderly (PASE) [31]. Weight and height were measured using a standard balance beam or digital scale, and height using a wall-mounted Harpenden stadiometer (Holtain, UK), respectively, to calculate body mass index (BMI). Comorbidities included any reported history of current or prior diabetes mellitus or cardiovascular disease. Insomnia was self-reported as having trouble getting to sleep or waking at night 3 times or more per week. Depression was defined as a score of > = 6 on the Geriatric Depression Scale [32]. The measure of sleep disordered breathing (SDB) used in this analysis was the apnea-hypopnea index (AHI). Apnea was defined as complete or near complete cessation of airflow for >10 seconds, and hypopneas were scored if clear reductions in breathing amplitude (at least 30% below baseline breathing) occurred, and lasted >10 seconds [33]. Only apneas and hypopneas that were each associated with 3% or greater desaturation were included in these analyses. AHI was calculated as the total number of apneas and hypopneas per hour of sleep. The inter-scorer reliability for AHI was high (ICC = 0.99).

### Statistical Analysis

The outcome variable in the current analysis was UFC. Predictor variables consisted of: (a) self-reported sleep duration; (b) actigraphic sleep duration; and (c) SWS from home PSG. The predictor variables were expressed categorically to account for any non-linear associations with the outcomes (sleep duration as ≤5 hours, >5 to <8 hours, >8 hours; SWS as quartiles). The cut-offs for categories of habitual sleep duration were chosen after assessing other possible categorizations. Baseline characteristics were summarized as means and standard deviations (SD) for continuous covariates, and counts and percentages for categorical data. These characteristics were compared across categories of the predictors, using ANOVA for continuous variables with normal distributions, Kruskal-Wallis tests for continuous variables with skewed distributions, and chi-square tests for categorical data.

Linear regression was used to assess the relationship of the predictor with the continuous outcome of 24-hour UFC. Tests for linear trend were performed by including the predictor variable as an ordinal variable in the models. Results were presented as adjusted means and 95% confidence intervals across quartiles or categories, calculated using the least-squares means procedure and a *P*-value test for a linear trend across categories. All models were first adjusted for age and then adjusted for other covariates. Potential confounding variables, identified *a priori* included race, BMI, physical activity, AHI, alcohol use, smoking status, history of diabetes, history of CVD, presence of depression, and sleep efficiency. Any covariate associated with the outcome or one or more predictors at a p<0.10 in univariate analyses was included in the multivariable model. These covariates were entered into sequential models, thereby assessing their incremental effects. The minimally adjusted model was adjusted for age, race and AHI. The multivariable adjusted model included age, race, AHI, as well as BMI, history of CVD, number of depressive symptoms, and physical activity. The models with actigraphic sleep duration and SWS as predictor variables were further adjusted for sleep efficiency.

The interaction of depression and insomnia and these sleep predictors was examined. To enable comparability to other studies of younger populations, the interactions of the predictors with age (<75 vs ≥75 years old) were explored. The interaction of the sleep predictors and history of CVD was also examined, due to the strong association of CVD with circadian rhythms (and, potentially, with cortisol) [34]. Stratifications by these parameters were performed where appropriate (when interaction p<0.10 in models adjusted for age, the predictor, stratification term and the interaction of the predictor and the stratification term).

Secondary analyses were conducted in the models with the predictors as a continuous variable; because the results did not significantly differ, only data with the predictor expressed as a categorical variable are presented. Sleep duration by PSG was also assessed as a predictor variable; because the results did not differ from actigraphic sleep duration, we report only the latter. Sensitivity analyses were performed using categorically defined depression rather than the number of depressive symptoms in the multivariable models. Sensitivity analysis was also performed in the 280 men who had actigraphy coinciding with the urine collection. For all models, mulitcollinearity of the variables were examined by calculating the variance inflation factor (VIF) for each variable. The maximum VIF was 1.17, indicating a low degree of multicollinearity. Regression diagnostics were performed on the data. All analyses were performed using SAS statistical software (version 9.2, SAS Institute, Inc., Cary, NC).

## Results

Table 1 summarizes the baseline characteristics of the participants included in this analysis. These subjects (n = 325) were similar to the larger MrOS Sleep Study cohort (n = 3104) with respect to age (77 vs. 76, p = 0.5), race/ethnicity (91% vs. 90% Caucasian, p = 0.14) and co-morbidities such as history of cardiovascular disease (31% vs. 33%, p = 0.3). The mean 24-hour UFC was 27.2 µg (SD 13.2), and average actigraphic sleep duration was 6.4 hours (SD 1.3).

**Table 1:** Baseline Characteristics.

Characteristic	(N = 325)
Age, mean ± SD, y	76.6±5.5
Non-Caucasian race, n (%)	29 (8.9)
Body mass index, kg/m^2^, mean ± SD	26.8±3.6
PASE score, mean ± SD	148.0±69.7
Apnea-hypopnea Index, mean ± SD	14.3±14.0
Insomnia, n (%)	193 (59.4)
Alcohol intake in past 12 months (drinks/week), n (%)	
0–2	169 (52.3)
3–13	133 (41.2)
≥14	21 (6.5)
Smoking, n (%)	
Never	135 (41.5)
Past	183 (56.3)
Current	7 (2.2)
History of diabetes mellitus, n (%)	42 (12.9)
History of CVD*, n (%)	99 (30.6)
Geriatric Depression Score (0–15), mean ± SD	1.7±2.1
GDS≥6 (depressed), n (%)	19 (5.9)
**Sleep Variables**	
Actigraphic sleep duration, hrs, mean ± SD	6.4±1.3
Actigraphic sleep categories, n (%)	
≤5 hrs	32 (10.5)
>5 to <8 hrs	250 (82.2)
≥8 hrs	22 (7.2)
Duration of slow wave sleep, min, mean ± SD	45.9±32.5
% of sleep time spent in slow wave sleep, mean ± SD	12.9±9.1
Self-reported sleep duration, mean ± SD	7.0±1.2
Self-reported sleep categories, n (%)	
≤5 hrs	29 (8.9)
>5 to <8 hrs	185 (56.9)
≥8 hrs	111 (34.2)
Sleep efficiency, %, mean ± SD	78.9±11.9
WASO, min, mean ± SD	73.9±39.8
24-hours urine free cortisol, µg, mean ± SD	27.2±13.2

* CVD includes history of myocardial infarction, angina, congestive heart failure, bypass surgery, angioplasty, or pacemaker placement.

The average of self-reported sleep duration was higher than average sleep estimated by actigraphy (7.0±1.2 hours vs. 6.4±1.3 hours, p<0.001, respectively). Approximately 9% of men slept less than 5 hours per night (self-report 8.9%, actigraphic 10.5%). The proportion of men with 8 or more hours of sleep per night differed between self-report and actigraphy (self-reported 34.2%, actigraphic 7.2%). There was only 54% agreement between the 2 measures across the categories, and the correlation of the linear representations was modest (r = 0.31). On average, those in the lower category of actigraphic sleep duration had higher levels of BMI and AHI and lower levels of sleep efficiency (p<0.05). Those in the lower category of self-reported sleep duration were less likely to be Caucasian and were more likely to report insomnia (p<0.05).

On average, the total duration of SWS was 46 minutes and accounted for 12% of sleep duration. SWS was significantly associated only with race. UFC was associated to physical activity and history of CVD.

**Figure 1:**
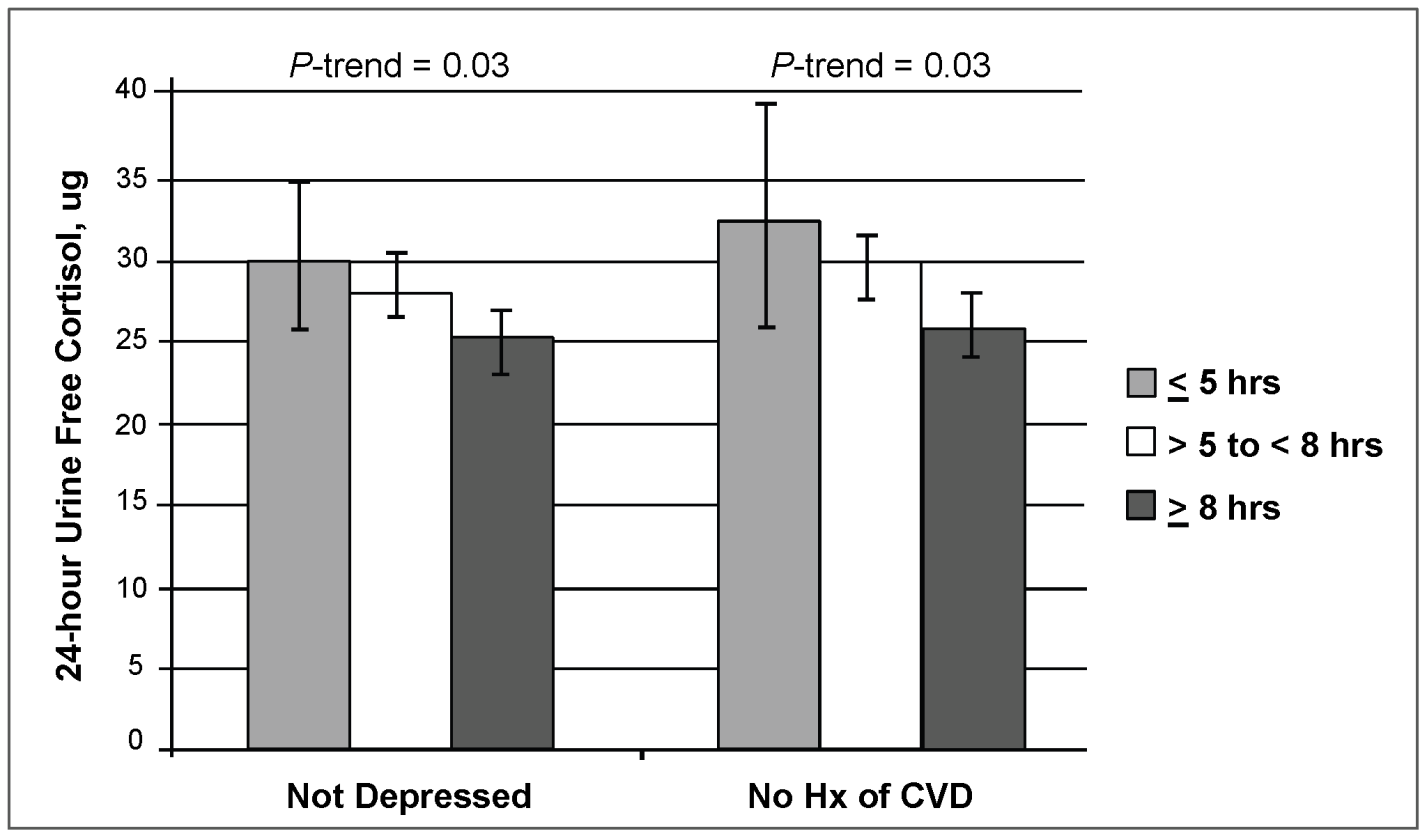
Association of self-reported sleep duration and 24-hour urine free cortisol among those without depression and those without a history of CVD. 24-hour urine free cortisol results are adjusted means (95% confidence intervals). Results for the not depressed sub-group adjusted for age, race, apnea-hypopnea index, BMI, history of CVD and physical activity. Results from those without a history of CVD adjusted for age, race, apnea-hypopnea index, BMI, physical activity, and number of depressive symptoms.

In the age-adjusted analysis (Table 2), there was an inverse association between self-reported sleep duration and 24-hour UFC (p = 0.08). In participants who reported 5 hours or less of habitual sleep, the adjusted mean 24-hour UFC was 29.2 µg, compared to 27.9 µg in those who reported >5 to <8 hours of sleep and 25.4 µg in those reporting 8 hours or more of sleep. In multivariable models adjusting for potential confounders, an inverse relationship was observed between self-reported sleep duration and 24-hour UFC (p = 0.058). Participants who reported 5 hours or less of habitual sleep had an adjusted mean 24-hour UFC of 29.8 µg (95% CI: 25.0, 34.7), compared to 28.0 µg (95% CI: 26.1, 29.8) in participants reporting >5 to <8 hours of sleep and 25.5 µg (95% CI: 23.1, 27.9) in those reporting 8 hours or more of sleep. Sensitivity models replacing the number of depressive symptoms with the presence of depression (as a covariate) in the multivariable models showed similar adjusted means and a slight improvement in statistical significance (*P*-trend = 0.047). Sensitivity analysis was also performed in the 280 men who had urine collection coinciding with actigraphy; the results in this subset were similar to that of the larger group. Restricting the analyses to participants without depression or a history of CVD showed a significant association between 24-hour UFC and self-reported sleep duration (*P*-trend = 0.03 for both models, Figure 1). Among those 306 men classified as not depressed (GDS<6) there is a linear trend across category of self-reported sleep duration, with those reporting <5 hours having an adjusted mean 24-hour UFC of 30.0 µg, and those with >8 hours of sleep duration having an adjusted mean value of 25.2 µg (*P*-trend = 0.03 and *P*-interaction = 0.09). While the interaction of history of CVD and self-reported sleep duration was not significant (*P*-interaction = 0.43), among those 225 men without a history of CVD there was a significant association of self-reported sleep duration and 24-hr UFC (*P*-trend = 0.03, Figure 1). There were no significant interactions between self-reported sleep duration and insomnia or age.

**Table 2:** Associations of Sleep Characteristics and 24-hour Urine Free Cortisol, µg, Adjusted Means (95% Confidence Intervals).

	Age	Minimally	Multivariable	Multivariable+ Sleep Fragmentation
Sleep Characteristic	Adjusted	Adjusted^a^	Adjusted^b^	Adjusted^c^
Self-Reported Sleep Duration				
≤5 hrs (n = 29)	29.2 (24.4, 34.0)	29.0 (24.2, 33.9)	29.8 (25.0, 34.7)	–
>5 to <8 hrs (n = 185)	27.9 (26.0, 29.8)	28.0 (26.1, 29.9)	28.0 (26.1, 29.9)	–
≥8 hrs (n = 111)	25.4 (23.0, 27.8)	25.5 (23.1, 28.0)	25.5 (23.1, 27.9)	–
*P*-trend	0.076	0.101	0.058	–
Actigraphic Sleep Duration				
≤5 hrs (n = 32)	24.9 (20.2, 29.5)	25.2 (20.4, 29.9)	25.1 (20.4, 29.8)	26.5 (20.5, 32.5)
>5 to <8 hrs (n = 250)	27.6 (25.9, 29.2)	27.7 (26.0, 29.4)	27.9 (26.2, 29.5)	27.7 (26.0, 29.4)
≥8 hrs (n = 22)	26.0 (20.4, 31.5)	25.7 (20.0, 31.3)	24.7 (19.2, 30.3)	24.1 (18.3, 29.9)
*P*-trend	0.63	0.76	0.92	0.55
% of Sleep time spent in SWS				
Q1:<5.06% (n = 79)	28.7 (25.8, 31.6)	28.6 (25.6, 31.6)	28.7 (25.8, 31.6)	28.5 (25.4, 31.5)
Q2∶5.06 to <11.90% (n = 80)	24.8 (21.9, 27.7)	24.8 (21.9, 27.7)	24.7 (21.9, 27.6)	24.7 (21.7, 27.7)
Q3∶11.90 to <18.90% (n = 79)	29.2 (26.3, 32.1)	29.3 (26.3, 32.2)	29.4 (26.5, 32.2)	29.8 (26.8, 32.8)
Q4: ≥18.90% (n = 80)	26.1(23.2, 28.9)	26.2 (23.3, 29.1)	26.2 (23.3, 29.1)	26.2 (23.2, 29.2)
*P*-trend	0.60	0.70	0.70	0.85

a Adjusted for age, race, apnea-hypopnea index.

b Adjusted for age, race, apnea-hypopnea index, BMI, history of CVD, physical activity, number of depression symptoms.

c Adjusted for age, race, apnea-hypopnea index, BMI, history of CVD, physical activity, number of depressive symptoms, sleep efficiency.

No significant associations were noted between 24-hour UFC and actigraphic sleep duration despite incremental adjustments for age, race, AHI, BMI, history of CVD, physical activity and number of depression symptoms (Table 2). Results were similar after further adjusting the multivariable models by sleep efficiency. There were no significant interactions of actigraphic sleep duration and age, depression, history of CVD or insomnia. Results were similar when replacing actigraphic sleep duration by PSG-measured sleep duration.

No significant association between SWS and 24-hour UFC was observed in any of models (Table 2). There were no significant interactions of SWS and age, depression, history of CVD or insomnia.

## Discussion

The primary finding of the current study is the lack of association between objectively measured (i.e. actigraphic) sleep duration and integrated cortisol levels in either unadjusted or adjusted analyses. Interestingly, we found an inverse association between self-reported sleep duration and 24-hour urinary cortisol levels even after adjusting for several confounding variables. The association was particularly strong in men without prevalent CVD and depression.

An emerging body of evidence indicates that short sleep duration may cause insulin resistance, glucose intolerance, and obesity [2]–[4]. However, the mechanisms by which this may occur remain unclear. Possible candidates include increase in corticotrophin activity, altered levels of circulating adipocytokines, decreased energy expenditure, and changes in activity levels. The majority of studies assessing cortisol levels have been performed in healthy volunteers under tightly controlled laboratory conditions with severe curtailment of total sleep time. While some studies have shown that cortisol levels are elevated [7], [8], others have shown that cortisol is unchanged or even decreased [9]–[11] as sleep duration in decreased. There are only two studies of community-based individuals in which the association between cortisol and sleep duration have been determined. In the Whitehall study of over 2700 men and women, Kumari et al found that self-reported short sleep duration was associated with a flatter diurnal slope of cortisol secretion and increased cortisol awakening response [12]. The Whitehall study used multiple salivary cortisol measurements taken over 1 day to provide an integrated measure of cortisol secretion, but sleep duration was based on self-report. A small (n = 90), community-based study found no association between sleep duration and morning salivary cortisol levels [13]. Although sleep duration was measured by actigraphy, the investigators assessed cortisol levels at a single time point. Due to the significant daily fluctuation of cortisol, single measurements are often unreliable [14].

The discrepancy we found between objective and subjective sleep in the analyses presented herein suggests a way to reconcile the findings of the other 2 community based studies. In this current analysis, both objective and subjective assessments of sleep duration are available within the same population. The correlation between self-reported and actigraphic sleep duration was modest overall (r = 0.31), and there was only 54% agreement between the 2 measures across categories. We found no association between objectively measured sleep duration and integrated cortisol levels, but did observe an inverse association between self-reported sleep duration and integrated cortisol levels. One possible explanation for this discrepancy is that self-reported sleep is a surrogate for other issues. For example, it is possible that those with more anxiety or “hyperarousal” report less subjective sleep. More generally, self-reported sleep time may be a marker for other social, psychological or physical issues. For example, self-reported sleep may be reflective of poor sleep quality due to depression, insomnia, or other factors. Lastly, self-reported sleep may be indicative of sleep duration over a longer period of time (i.e., weeks to months), whereas actigraphy measured sleep duration over 5 days (in this cohort).

Studies that have assessed the association between objective and subjective sleep duration have had similar findings regarding the discordance between objective and subjective sleep duration [35], [36].

Lauderdale et al found that subjective sleep measurement was greater than objective sleep duration due, most likely, to inaccurate reporting of how much an individual sleeps on a single night [35]. In a small study (n<100) of post-menopausal women, Regestein et al found a similar discrepancy between self-reported and actigraphic sleep times [36]. Interestingly, Regestein et al found that high hyperarousal scores (on a Hyperarousal Scale) were associated with ***underestimations*** of objective sleep; participants in the lowest hyperarousal score quartile ***overestimated*** their objective sleep. The investigators also found that increased psychological and somatic symptoms were associated with self-reported low sleep quality.

The current study has several important limitations that warrant consideration. First, the MrOS cohort was older in age, male, and predominantly Caucasian, thereby limiting the generalizability of our findings. Second, the 24-hour urine collection was performed at home, not in a laboratory, so the accuracy of the collection cannot be absolutely determined. The strengths of this study include the use of both actigraphic and self-reported sleep data within the same population, allowing for the comparison of the two. Furthermore, cortisol was measured using an integrated measure of secretion (24-hour UFC), rather than a single time point, which is highly unreliable due to its daily variability. Finally, the fact that participants were in their usual environment (rather than a laboratory setting) allows for a more realistic assessment of their sleep and possibly of their cortisol secretion. In conclusion, we found that objectively measured sleep duration was not associated with integrated cortisol levels in this cohort of community dwelling men. This finding, together with prior studies, suggest that elevated levels of integrated cortisol secretion are not the mechanism by which short sleep duration leads to adverse health outcomes. Further studies will need to be done to confirm these findings in other groups, including those that are young and female.

## Supporting Information

Acknowledgments S1 (DOC)

## References

[1] National Sleep Foundation (2011) “Sleep in America” poll. Washington DC: National Sleep Foundation.

[2] Kripke DF, Simons RN, Garfinkel L, Hammond EC (1979) Short and long sleep and sleeping pills. Is increased mortality associated? Arch Gen Psychiatry 36: 103–116. 760693 10.1001/archpsyc.1979.01780010109014

[3] Spiegel K, Leproult R, Van Cauter E (1999) Impact of sleep debt on metabolic and endocrine function. Lancet 354: 1435–1439. 10543671 10.1016/S0140-6736(99)01376-8

[4] Cappuccio FP, Taggert FM, Kandala N, Currie A, Peile E, et al (2008) Meta-analysis of short sleep duration and obesity in children and adults. Sleep 31: 610–626. 10.1093/sleep/31.5.619 PMC2398753 18517032

[5] Spiegel K, Sheridan JF, Van Cauter E (2002) Effect of sleep deprivation on response to immunization. JAMA 288: 1471–1472. 10.1001/jama.288.12.1471-a 12243633

[6] Patel SR, Blackwell T, Redline S, Ancoli-Israel S, Cauley JA, et al (2008) The association between sleep duration and obesity in older adults. Int J Obes 32: 1825–1834. 10.1038/ijo.2008.198 PMC2605208 18936766

[7] Leproult R, Copinschi G, Buxton O, Van Cauter E (1997) Sleep loss results in an elevation of cortisol levels the next evening. Sleep 20: 865–870. 9415946

[8] Chapotot F, Buguet A, Gronfier C, Brandenberger G (2001) Hypothalamo-pituitary-adrenal axis activity is related to the level of central arousal: effect of sleep deprivation on the association of high-frequency waking electroencephalogram with cortisol release. Neuroendocrinology 73: 312–321. 11399904 10.1159/000054648

[9] Follenius M, Brandenberger G, Bandesapt JJ, Libert JP, Ehrhart J (1992) Nocturnal cortisol release in relation to sleep structure. Sleep 15: 21–27. 1557591 10.1093/sleep/15.1.21

[10] Dinges DF, Douglas SD, Zaugg L, Campbell DE, McMann JM, et al (1994) Leukocytosis and natural killer cell function parallel neurobehavioural fatigue induced by 64 hours of sleep deprivation. J Clin Invest 93: 1930–1939. 7910171 10.1172/JCI117184 PMC294300

[11] Kant GJ, Genser SG, Rhorne DR (1984) Effects of 72 hour sleep deprivation on urinary cortisol and indices of metabolism. Sleep 7: 142–146. 6740058 10.1093/sleep/7.2.142

[12] Kumari M, Badrick E, Ferrie J, Perski A, Marmot M, et al (2009) Self-reported sleep duration and sleep disturbance are independently associated with cortisol secretion in the Whitehall II study. J Clin Endocrinol Metab 94: 4801–4809. 19850688 10.1210/jc.2009-0555 PMC2795654

[13] Zhang J, Ma RCW, Kong APS, So WY, Li AM, et al (2011) Relationship of sleep quantity and quality with 24-hour urinary catecholamines and salivary awakening cortisol in healthy middle-aged adults. Sleep 34: 225–233. 21286244 10.1093/sleep/34.2.225 PMC3022944

[14] Stewart J and Seeman T (1999) Salivary Cortisol Measurement. Summary of Conference organized by the MacArthur Research Network on SES and Health at Rockefeller University. Available: www.macses.ucsf.edu. Accessed 6 Mar 2013.

[15] Contreras LN, Hane S, Tyrrell JB (1986) Urinary cortisol in the assessment of pituitary-adrenal function: utility of 24-hour and spot determinations 1986. J Clin Endocrinol Metab 62: 965–969. 3958132 10.1210/jcem-62-5-965

[16] Nicolson NA (2008) Measurement of Cortisol. In: Luecken LJ, Gallo LC, editors. Handbook of Physiological Research Methods in Health Psychology. Thousand Oaks:Sage Publications.

[17] Gatti R, Antonelli G, Prearo M, Sinella P, Cappellin E, et al (2009) Cortisol assays and diagnostic laboratory procedures in human biological fluids. Clinical Biochemistry 42: 1205–1217. 19414006 10.1016/j.clinbiochem.2009.04.011

[18] Orwoll E, Blank JB, Barrett-Connor E, Cauley J, Cummings S, et al. 2005 Design and baseline characteristics of the osteoporotic fractures in men (MrOS) study–a large observational study of the determinants of fracture in older men. Contemp Clin Trials 26: 569–585. 16084776 10.1016/j.cct.2005.05.006

[19] Blank JB, Cawthon PM, Carrion-Petersen ML, Harper L, Johnson JP, et al. 2005 Overview of recruitment for the osteoporotic fractures in men study (MrOS). Contemp Clin Trials 26: 557–568. 16085466 10.1016/j.cct.2005.05.005

[20] Aron DC, Findling JW, Tyrrell JB (2004) Glucocorticoids and Adrenal Androgens. In: Greenspan FS, Gardner DG, editors. Basic and Clinical Endocrinology. New York:McGraw-Hill Companies. 362–410.

[21] Nieman LK, Biller BMK, Findling JW, Newell-Price J, Savage MO, et al (2008) The diagnosis of Cushing’s syndrome: an Endocrine Society clinical practice guideline. J Clin Endocrinol Metab 93: 1526–1540. 18334580 10.1210/jc.2008-0125 PMC2386281

[22] Brown PH, Blundell G, Greening AP, Crompton GK (1991) Screening for hypothalamo-pituitary-adrenal axis suppression in asthmatics taking high dose inhaled corticosteroids. Respiratory Medicine 85: 511–516. 1775678 10.1016/s0954-6111(06)80269-6

[23] Ancoli-Israel S, Cole R, Alessi C, Chambers M, Moorcroft W, et al (2003) The role of actigraphy in the study of sleep and circadian rhythms. Sleep 26: 342–392. 12749557 10.1093/sleep/26.3.342

[24] Action-W User’s Guide, Version 2.0. Ambulatory Monitoring, Inc. Ardsley NY.

[25] Blackwell T, Ancoli-Israel S, Gehrman PR, Schneider JL, Pedula KL, et al (2005) Actigraphy scoring reliability in the study of osteoporotic fractures. Sleep 28: 1599–1605. 16408420 10.1093/sleep/28.12.1599

[26] Jean-Louis G, Kripke DF, Mason WJ, Elliot JA, Youngstedt SD (2001) Sleep estimation from wrist movement quantified by different actigraphic modalities. J Neurosci Methods 105: 185–191. 11275275 10.1016/s0165-0270(00)00364-2

[27] Blackwell T, Redline S, Ancoli-Israel S, Stone KL (2007) Comparison of total sleep time from actigraphy and polysomnography in older men. The MrOS Sleep Study. Sleep 30: 346–347.

[28] Redline S, Sanders MH, Lind BK, et al (1998) Methods for obtaining and analyzing unattended polysomnography data for a multicenter study: Sleep Heart Health Research Group. Sleep 21: 759–767. 11300121

[29] Rechtschaffen A, Kales A (1968) A manual of standardized terminology, techniques and scoring system for sleep stages of human subjects. Washington DC: National Institutes of Health. NIH Publication 204.

[30] Rao MN, Blackwell T, Redline S, Stefanick ML, Ancoli-Israel S, et al (2009) Association between sleep architecture and measures of body composition. Sleep 32: 483–490. 19413142 10.1093/sleep/32.4.483 PMC2663862

[31] Washburn RA, Ficker JL (1999) Physical Activity Scale for the Elderly (PASE): the relationship with activity measured by a portable accelerometer. J Sports Med Phys Fitness 39: 336–340. 10726435

[32] Sheikh J, Yesavage J (1986) Geriatric Depression Scale: recent evidence and development of a shorter version. In: Brink TL, editor. Clinical Gerontology : A Guide to Assessment and Intervention. New York: The Haworth Press. 165–173.

[33] Quan SF, Wright R, Baldwin CM, Kaemingk KL, Goodwin JL, et al (2006) Obstructive sleep apnea-hypopnea and neurocognitive functioning in the Sleep heart Health Study. Sleep Med 7: 498–507. 16815753 10.1016/j.sleep.2006.02.005

[34] Muller JE, Stone PH, Zoltan GT, et al (1985) Circadian variation in the frequency of onset of acute myocardial infarction. N Engl J Med 313: 1315–1322. 2865677 10.1056/NEJM198511213132103

[35] Lauderdale DS, Knutson KL, Yan LL, Liu K, Rathouz PJ (2008) Sleep duration: how well do self-reports reflect objective measures? The CARDIA Sleep Study. Epidemiology 19: 838–845. 18854708 10.1097/EDE.0b013e318187a7b0 PMC2785092

[36] Regestein QR, Friebely J, Shifren JL, Scharf MB, Wilita B, et al (2004) Self-reported sleep in postmenopausal women. Menopause 11: 198–207. 15021450 10.1097/01.gme.0000097741.18446.3e

