# Supplementary material for: Association between Sleep Duration and 24-Hour Urine Free Cortisol in the MrOS Sleep Study
Source: PLoS One. 2013 Sep 27;8(9):e75205. doi: 10.1371/journal.pone.0075205 (PMC3815404; doi:10.1371/journal.pone.0075205)
Supplement: Acknowledgments S1 — (DOC) [file pone.0075205.s001.doc]

Acknowledgments

Investigators in the Outcomes of Sleep Disorders in Older Men study (MrOS Sleep):

***Coordinating Center (California Pacific Medical Center Research Institute and University of California, San Francisco)*:** K.L. Stone (Principal Investigator), D.C. Bauer (co-Investigator), S.R. Cummings (co-Investigator), N. Goldschlager (co-Investigator), G. Tranah (co-Investigator), P. Varosy (co-Investigator), K. Yaffe (co-Investigator), P.M. Cawthon (co-Investigator), R. Fullman (Project Director), R. Benard, T. Blackwell, L. Concepcion, J. Diehl, S. Ewing, C. Fox, M. Jaime-Chavez, E. Kwan, S. Litwack, W. Liu, L.Y. Lui, K. Peters, W. Sauer, J. Schneider, R. Scott, D. Tanaka, J. Ziarno; ***Administrative Center (Oregon Health & Sciences University):*** E. Orwoll (Principal Investigator), C. Lee (co-Investigator), C. Pedersen (Project Director), M. Abrahamson, L Masterfield; ***University of Alabama, Birmingham:*** C.E. Lewis (Principal Investigator), J. Shikany (co-Investigator), P. Johnson (Project Director), M. Young, S. House, N. Webb, S. Felder, J. King, T. Johnsey, C. Collier, K. Hardy, J. Smith, H. Dwivedi; ***University of Minnesota*:** K. Ensrud (Principal Investigator), S. Diem (co-Investigator), H. Fink (co-Investigator), N. Nelson (Clinic Coordinator), R. Andrews, S. Fillhouer, M. Forseth, K Jacobson, S. Luthi, K. Moen, M. Paudel, P. Van Coevering, S. Ziesche*;* ***Stanford University***: M. Stefanick (Principal Investigator), A. Hoffman (co-Investigator), K. Kent, N. Ellsworth, S. Belding, A. Krauss; ***University of Pittsburgh*:** J. Cauley (Principal Investigator), J. Zmuda (co-Investigator), M. Danielson (Study Administrator), L. Harper (Project Director), L. Buck (Clinic Coordinator), D. Cusick, M. Gorecki, C. Newman; ***University of California, San Diego*:** E. Barrett-Connor (Principal Investigator), S. Ancoli-Israel (co-Investigator), T. Dam (co-Investigator), ML Carrion-Petersen (Project Director), D. Claflin, N. Kamantigue, K. Marksbury Jappe, P. Miller, M. Stephens; ***Brigham and Women's Hospital Sleep Reading Center:*** S. Redline (Principal Investigator), S. Surovec (Project Administrator), D. Mobley (Chief Polysomnologist), M. Rueschman (Programmer Analyst), M. Morrical (Polysomnologist), J. Arnold (Polysomnologist), R. Nawabit (Polysomnologist).
